# People Internalized More Attitudes Toward Aging During the Pandemic

**DOI:** 10.1093/geroni/igab046.2304

**Published:** 2021-12-17

**Authors:** Helene Fung, Hiu Ling Vivian Tsang, Chunyan Mai

**Affiliations:** 1 The Chinese University of Hong Kong, Shatin, N.T., Hong Kong, Hong Kong; 2 Hong Kong Metropolitan University, The Chinese University of Hong Kong, Hong Kong; 3 the Chinese University of Hong Kong, Hong Kong, Hong Kong

## Abstract

Older adults are viewed as being vulnerable to COVID-19. Previous research revealed that individuals would internalize or dissociate with attitudes toward aging when they aged. In this study, data collected before the COVID-19 pandemic were compared with those collected during the pandemic to assess whether the pandemic might make older adults internalize or dissociate with attitudes toward aging to a greater extent.123 Hong Kong participants (50.4% females, M=60.59±13.28 years old) were recruited in a two-wave survey (wave 1 in 2018 and wave 2 in 2020) on attitudes toward aging and future self-views. After comparing the correlations between attitudes toward aging and future self-views in the two waves, we found stronger positive correlations between these 2 variables in wave 2 than in wave 1 in the personality and finance domains, but not in the family, independence, or health domains. These findings suggest that internalization of attitudes toward aging might be domain-specific. The pandemic might make older adults more likely to internalize positive personality attitudes toward aging and negative finance attitudes toward aging into their future self-views. Professionals may consider utilizing the internalization process to promote a positive attitude toward aging during the pandemic.

